# Human Macrophages Preferentially Infiltrate the Superficial Adipose Tissue

**DOI:** 10.3390/ijms19051404

**Published:** 2018-05-08

**Authors:** Giuseppe Cappellano, Evi M. Morandi, Johannes Rainer, Philipp Grubwieser, Katharina Heinz, Dolores Wolfram, David Bernhard, Susanne Lobenwein, Gerhard Pierer, Christian Ploner

**Affiliations:** 1 Department of Plastic, Reconstructive and Aesthetic Surgery, Medical University of Innsbruck, Anichstrasse 35, 6020 Innsbruck, Austria; giuseppe.cappellano@i-med.ac.at (G.C.); evi.morandi@tirol-kliniken.at (E.M.M.); philipp.grubwieser@student.i-med.ac.at (P.G.); dolores.wolfram@i-med.ac.at (D.W.); susanne.lobenwein@i-med.ac.at (S.L.); gerhard.pierer@i-med.ac.at (G.P.); 2 Institute for Biomedicine, Eurac Research, Affiliated Institute of the University of Lübeck, Viale Druso 1, 39100 Bolzano, Italy; johannes.rainer@eurac.edu; 3 Cardiac Surgery Research Laboratory, University Clinic for Cardiac Surgery, Medical University of Innsbruck, 6020 Innsbruck, Austria; katharina.heinz@i-med.ac.at; 4 Center for Medical Research, Medical Faculty, Johannes-Kepler-University Linz, 4020 Linz, Austria; david.bernhard@jku.at

**Keywords:** adipose-derived stem cells, superficial adipose tissue, deep adipose tissue, immune cell infiltration, macrophages

## Abstract

Human abdominal subcutaneous adipose tissue consists of two individual layers—the superficial adipose tissue (SAT) and deep adipose tissue (DAT)—separated by the Scarpa’s fascia. The present study focuses on the analysis of morphological and immunological differences of primary adipocytes, adipose-derived stem cells (ASC), and tissue-infiltrating immune cells found in SAT and DAT. Adipocytes and stromal vascular fraction (SVF) cells were isolated from human SAT and DAT specimens and phenotypically characterized by in vitro assays. Ex vivo analysis of infiltrating immune cells was performed by flow cytometry. Primary adipocytes from SAT are larger in size but did not significantly differ in cytokine levels of LEPTIN, ADIPOQ, RBP4, CHEMERIN, DEFB1, VISFATIN, MCP1, or MSCF. ASC isolated from SAT proliferated faster and exhibited a higher differentiation potential than those isolated from DAT. Flow cytometry analysis indicated no specific differences in the relative numbers of ASC, epithelial progenitor cells (EPC), or CD3^+^ T-cells, but showed higher numbers of tissue-infiltrating macrophages in SAT compared to DAT. Our findings suggest that ASC isolated from SAT have a higher regenerative potential than DAT-ASC. Moreover, spatial proximity to skin microbiota might promote macrophage infiltration in SAT.

## 1. Introduction

Adipose tissue function and characteristics depend on the anatomical localization of the fat depot. Differences of subcutaneous adipose tissue and visceral adipose tissue (VAT) have been well-elaborated and a correlation concerning VAT volume and the risk to develop diabetes, coronary heart disease, and atherosclerosis have been described [1,2,3]. However, little is known about the morphological and functional differences within the same fat depot. This is particularly interesting for the abdominal subcutaneous fat tissue as it is divided in different layers rather than being a homogenous layer. Learning from murine subcutaneous fat tissue architecture, where the subcutaneous fat layer consists of three layers [4], the human abdominal subcutaneous adipose tissue consists of only two individual layers—the superficial adipose tissue (SAT) and deep adipose tissue (DAT)—separated by a membranous layer called Scarpa’s fascia [4]. Recent studies acknowledging this anatomical difference described depot-specific differences in adipocyte morphology and paracrine activity as well as a distinct regenerative potential for each of the two individual fat layers. From the morphological point of view, the tissue architecture of SAT is characterized by defined regular cuboid fat lobules encompassing single adipocytes and conferring SAT robustness against external mechanical cues. In contrast, DAT fat lobules are smaller, flat-shaped, and more irregular in size while being surrounded by a higher amount of connective tissue [5]. As DAT is superficially constrained by the Scarpa’s fascia and dorsally confined by the muscular abdominal wall, the purpose of this fat tissue might be different in acting more as a friction-bearing layer between the SAT and muscle, which is able to stretch and slide in response to external force [5]. The Scarpa’s fascia separating SAT and DAT is a clearly defined anatomical structure, which can be identified easily by sonography and magnetic resonance (MR) imaging [6]. Underlining the clinical importance, recent studies showed that a disproportionate accumulation of DAT (but not SAT) correlates with impaired systemic metabolism [7] similar to the well-investigated connection of high accumulation of VAT and various metabolic changes [8,9]. In line with these observations, DAT but not SAT showed a strong relation to insulin resistance and association with common features of metabolic diseases such as hypertension, cholesterol, or triglyceride levels [10,11].

These disease-promoting factors obviously correlate with the endocrine and paracrine activity of the adipose tissue depots and this activity is—at least in part—controlled by tissue infiltration and resident immune cells, such as T-cells and adipose-tissue-macrophages. Tissue infiltration by these cells might contribute to changes in adipokine levels and thus be responsible for disease progress. Moreover, the intrinsic metabolism regulates the fate of specific cell subsets in adipose tissue [12]. Indeed, a deep understanding of the cross-talk between adipose tissue and immune cells (referred to as “immunometabolism”) is important to develop new strategies to treat metabolic disorders.

Thus, we studied biological function and cellular tissue infiltrate composition of the different human subcutaneous adipose tissue depots in samples of post bariatric patients compared with peripheral blood samples of the same individuals. Our findings showed an increased proliferation and differentiation potential of adipose-derived stem cells (ASC) obtained from SAT over DAT and suggested that the amount of tissue infiltrating macrophages decreases with the distance to the dermal layer.

## 2. Results

### 2.1. Morphology and Paracrine Activity of SAT and DAT

**Figure 1:**
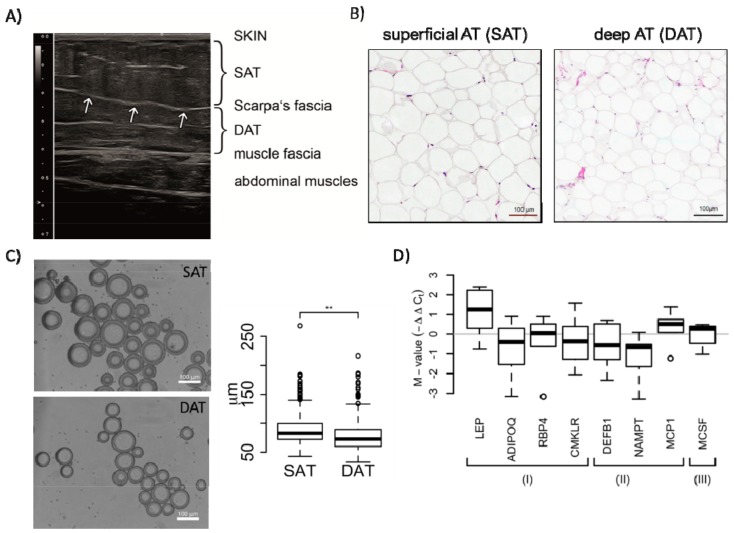
Morphological and paracrine characterization of superficial adipose tissue (SAT) and deep adipose tissue (DAT) adipocytes. (**A**) Representative ultrasound image of infraumbilical subcutaneous fat tissue showing the two individual subcutaneous fat layers. The arrows indicate the Scarpa’s fascia. Obviously, a higher level of hyperechogenic connective tissue structures was observed in DAT indicating structural fat tissue architecture and functional differences; (**B**) images of H&E-stained SAT and DAT cross-sections; (**C**) microscopy and quantitative analyses of freshly isolated adipocytes from SAT or DAT. The box plot represents data from a total of 2167 analysed adipocytes isolated from 3 female patients (Student’s *t*-test, ** *p*-values < 0.01); (**D**) RNA from SAT and DAT adipocytes was analysed for the expression of depicted cytokines by quantitative real time PCR. Expression values of indicated cytokines from six patients were normalized to the mean of three reference genes (GUSB, 18sRNA, and GAPDH) and are grouped according their function: (I) represents adipokines, (II) cytokines involved in inflammation and pathogen defence, (III) cytokine associated with neoangiogenesis. Shown are distributions of M-values (log2 fold-change values representing differential expression between SAT and DAT). Significance for difference of the means was calculated using a paired *t*-test.

Following our hypothesis that superficial and profound layers of the abdominal subcutaneous fat tissue exhibit profound morphological and functional differences, we applied high-resolution ultrasound to identify differences in tissue architecture and morphology (Figure 1A). In the presented image, SAT is separated by the clearly visible Scarpa’s fascia (arrows, Figure 1A) from the underlying profound DAT. Moreover, DAT tissue architecture was clearly discriminative from SAT, since DAT showed an increased amount of hyperechogenic connective tissue.

In addition, we characterized freshly isolated adipocytes from SAT and DAT layers regarding their morphology (size) and their paracrine activity (Figure 1B–D). First, we determined the size (diameter) of isolated adipocytes from SAT and DAT by software-based analysis of microscopy images (Figure 1C). These analyses showed that the size of adipocytes isolated from SAT significantly exceeded those from DAT, even if the adipocyte size in general varied between patients (Figure 1C and Figure S1).

To assess paracrine differences of the two subcutaneous fat layers, we analysed mRNA expression levels of “classical” adipokines, such as ADIPOQ, LEPTIN, and CHEMERIN (CMKLR), as well as cytokines that correlate with increased inflammation (DEFB1, VISFATIN (NAMPT), and MCP1) or angiogenesis (MCSF) in SAT and DAT by quantitative real time PCR. Among the investigated adipokines, we found that only LEPTIN was upregulated in SAT (*p*-value = 0.075). Among the inflammatory cytokines, MCP-1 was upregulated in SAT (*p*-value = 0.073), while DEFB1 and VISFATIN were downregulated, though not reaching statistical significance due to interdonor variability (Figure 1D).

### 2.2. ASC from SAT Proliferate Faster and Have a Higher Differentiation Potential

**Figure 2:**
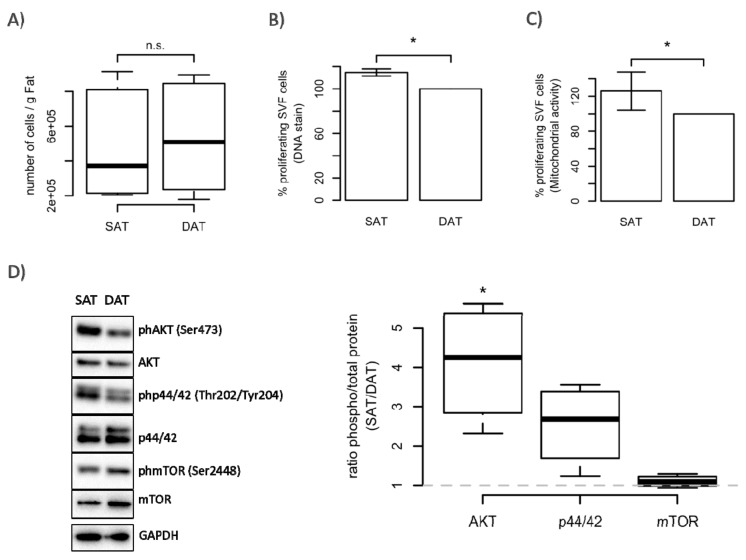
Stromal vascular fraction (SVF) cellularity and proliferation capacity of SAT- and DAT-derived adipose-derived stem cells (ASC). (**A**) Cellularity was calculated by correlating the numbers of SVF cells with the amount (g) of processed fat tissue. Data are shown as mean ± SD (*n* = 6); (**B**,**C**) proliferation of SAT and DAT ASC was assessed after culture for 6 days by analysing DNA content (CyQANT) and mitochondrial activity (PrestoBlue). Results are shown as % of DAT (set to 100%) from six patients; (**D**) representative immunoblot and quantitative assessment of day 3 proliferating ASC from four donors, analysing expression and phosphorylation of protein kinase B (AKT), extracellular signal-regulated kinase ERK 1/2 (p44/42), mammalian target of rapamycin (mTOR), and GAPDH as loading control. Data are shown as mean ± SD. Significance for difference of the means was calculated using a paired *t*-test (* *p*-value < 0.05).

**Figure 3:**
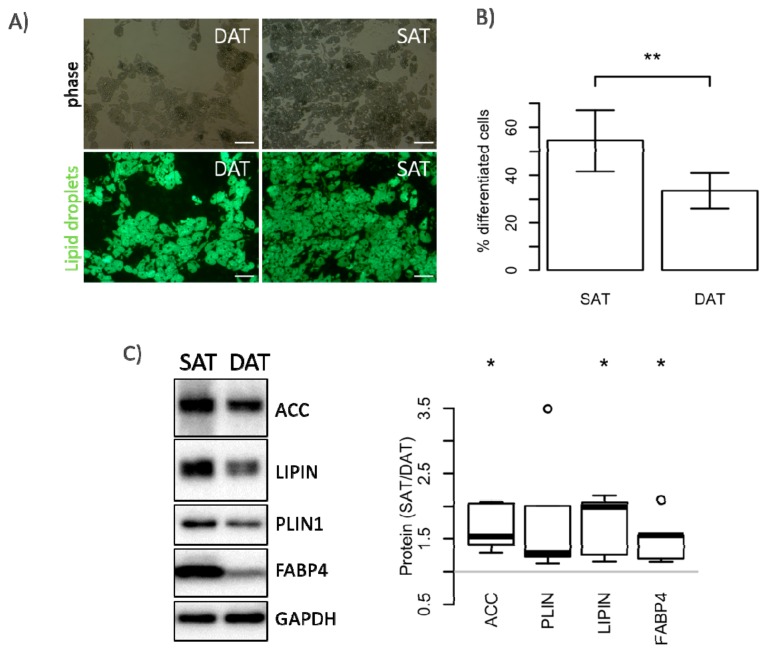
Adipocyte differentiation potential of SAT- and DAT-derived ASC. ASC isolated from SAT and DAT were differentiated in vitro for 14 days. BODIPY™ 493/503-stained adipocytes were analysed by fluorescence microscopy (**A**) and quantitatively assessed by flow cytometry (**B**); Size bar: 100 µm. Data are shown as mean ± SD (*n* = 6), significance for difference of the means calculated with a paired *t*-test (** *p*-value < 0.01); (**C**) Representative immunoblot and quantitative assessment of day 14 differentiated ASC from five donors, analysing expression of acetyl-CoA carboxylase (ACC), lipid-droplet-binding protein perilipin 1 (PLIN1), phosphatidate phosphatase lipin 1 (LPIN1), fatty acid transport protein 4 (FABP4), and GAPDH as loading control. Results are shown as box plots representing the distribution of fold change values; significance of the fold change was assessed by testing against the null hypothesis of a mean fold change of 1 (* *p*-value < 0.05).

We isolated ASC from the stromal vascular fraction (SVF) specific for each fat tissue depot and determined their proliferation and differentiation potential. Although we did not observe differences in the yield of isolated cells per gram of fat tissue (Figure 2A), ASC isolated from SAT (SAT-ASC) proliferated much faster than those isolated from DAT as shown in Figure 2B,C. These differences were also confirmed on the molecular level. In fact, SAT-ASC exhibited higher levels of the extracellular signal-regulated kinases ERK 1/2 (p44/42) and PI3-kinase controlled phosphorylation of AKT (Figure 2D). In addition, SAT-ASC differentiated more efficiently into adipocytes in vitro than those isolated from DAT (Figure 3A,B). In SAT-ASC, the number of lipid-droplet-containing cells was about 1/3 higher than in in vitro differentiated DAT-ASC (mean ± SD, mean SAT-ASC diff = 54.4% ± 12.8 versus mean DAT-ASC diff = 33.5% ± 7.4). These results were also confirmed by Western blot analysis, which showed that protein levels of the fatty acid synthase regulator acetyl-CoA carboxylase (ACC), lipid-droplet-binding protein perilipin 1 (PLIN1), phosphatidate phosphatase lipin 1 (LPIN1), and the fatty acid transport protein 4 (FABP4) in in vitro differentiated SAT-ASC exceeded those in differentiated DAT-ASC (Figure 3C). The observed differences in proliferation and differentiation could not be explained by different tissue cellularity, as SVF numbers per gram of fat tissue were not significantly different in SAT and DAT. 

### 2.3. Numbers of ASC Do Not Differ in SAT and DAT

**Figure 4:**
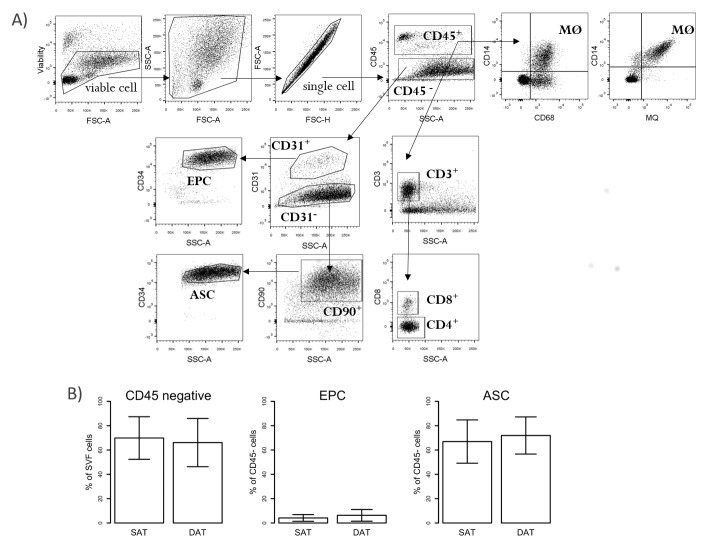
Flow cytometry analysis of ASC, endothelial progenitor cells (EPC), and T-cells in SVF from SAT, DAT, and blood. SVF cells isolated from SAT and DAT as well as peripheral blood mononuclear cells (PBMC) from paired samples were analysed by flow cytometry. (**A**) Representative FACS plots showing the gating strategy of different cell populations investigated in this study (FSC-A: forward scatter area; SSC-A: side scatter area; FSC-H: forward scatter height); (**B**) percentages of CD45^+^ and CD45^−^ are shown on viable cells. For further analysis, the percentages of cells were calculated based on CD45^+^ and CD45^−^ cells, respectively. EPC (CD45^−^CD31^+^CD34^+^) and ASC (CD45^−^CD31^−^CD90^+^CD34^+^) are shown as percentage (%) of CD45^−^ cells. Results represent data from five patients and are expressed as mean ± SD.

We applied flow cytometry to address the question of whether SAT or DAT contain different amounts of ASC, which might explain the increased proliferation and differentiation potential in SAT. Using CD45^−^CD31^−^CD90^+^CD34^+^ as markers to define CD34^+^ ASC within the SVF, we did not find significant differences in cell numbers of these populations in SAT and DAT. Similarly, the frequency of CD45^−^CD31^+^CD34^+^ endothelial progenitor cells (EPC), which might represent an alternative source of proliferating cells in the SVF, showed no difference (Figure 4A,B).

### 2.4. Numbers of SAT-Homing Macrophages Exceeded Those of DAT

**Figure 5:**
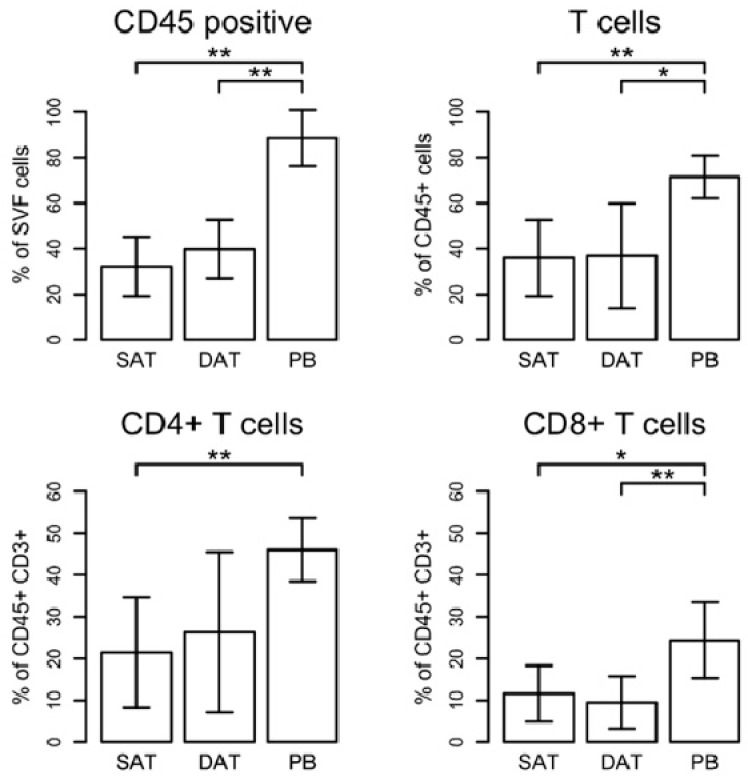
Analysis of T-cells in SAT, DAT, and peripheral blood cells (PB). Gating strategy is shown in Figure 4A. The percentages of T-cells were calculated based on the numbers of CD45^+^ cells. CD8^+^ T-cells were discriminated from CD4^+^ T-helper cells on the basis of expression of CD8 marker. CD4^+^ T-cells were determined as CD8^-^ cells. Results represent data from six patients and are expressed as mean ± SD. Significance was assessed using a paired *t*-test (* *p*-value < 0.05, ** *p*-value < 0.01).

Thus, we hypothesized that another cell subtype of the CD34^+^ ASC or interaction of those cells with infiltrating CD45^+^ immune cells might have affected ASC already in vivo, which committed them for faster proliferation and differentiation. To assess whether the amount of fat tissue infiltrating immune cells differs in the two subcutaneous layers, we analysed the frequency of CD45^+^ cells in the SVF. Overall, the percentages of these cells, which represent the global leucocyte cell population, did not vary between SAT and DAT specimens but they were significantly lower when compared with peripheral blood cells (PB) (Figure 5). CD45^+^ cells were further analysed to determine the frequencies of CD4^+^ T-Helper cells (CD45^+^CD3^+^CD4^+^), cytotoxic CD8^+^ T-cells (CD45^+^CD3^+^CD8^+^), and mature macrophages (CD45^+^CD68^+^CD14^+^). As shown in Figure 5, CD3^+^ T-cells infiltrate both SAT and DAT at comparable levels. The amount of CD3^+^ T-cells within CD45^+^ cells was 35.93% ± 6.88 (mean ± SEM) in SAT and 36.81% ± 9.39 in DAT, respectively, showing no substantial difference between the depots. Moreover, the frequency of CD3^+^ T-cells in SAT and DAT was significantly decreased in comparison with PB (71.53% ± 3.85), whereas the CD4^+^/CD8^+^ ratio did not change (Figure 5).

**Figure 6:**
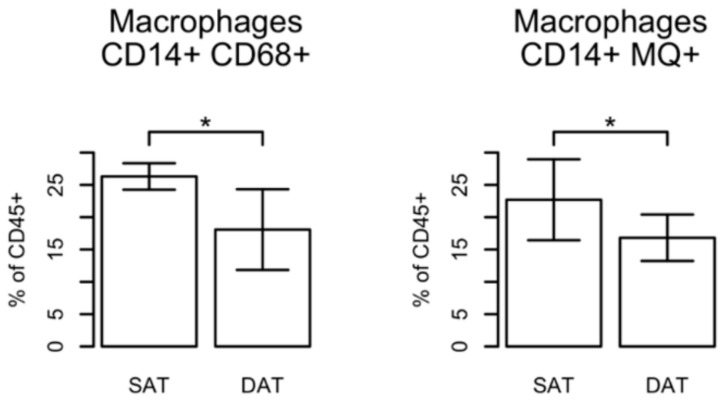
Macrophage infiltration in SAT and DAT. Gating strategy is shown in Figure 4A. Macrophages (defined as CD14^+^CD68^+^ or CD14^+^ MΦ^+^ (clone 25f9)) are shown as % of CD45^+^ cells. Results represent data from four patients and are expressed as mean ± SD. Significance of the difference in means was calculated using a paired *t*-test (* *p*-value < 0.05).

On the contrary, we found that in general the numbers of macrophages infiltrating the subcutaneous fat tissue (SCAT) were significantly increased in comparison with circulating macrophages in PB, and—even more interesting—a significant increase in the amount of macrophages in SAT compared with DAT (Figure 6 and Figure S2). We observed about 1.5-fold (%SAT/DAT, SAT = 26.3 ± 0.91 versus DAT = 18.1 ± 2.8) more mature macrophages in the fat tissue being localized more superficially near the dermal layer (SAT) and about 2.3-fold more (%SAT/PB, SCAT = 23.0 ± 1.8 versus DAT = 9.8 ± 3.2) in comparison to PB (Figure S2).

CD68 and CD14 markers were chosen as commonly used markers for human macrophages. Taking into account that both markers can also be expressed by monocytes or—in case of CD68—also in non-immune cells, such as fibroblasts [13], we confirmed our observations by staining the cells with a tissue macrophage marker (MQ, clone 25f9), which has been shown to be specific for mature macrophages and is not found on monocytes [14]. Similar to our previous findings using CD68 marker, we observed a significant increase of the amount of MQ^+^ macrophages in SAT in comparison with DAT (Figure 6). Interestingly, when the MQ and CD14 markers were used in combination, we found that MQ^+^ cells were positive for CD14 in all donors, whereas the staining using a combination of anti-CD68 and anti-CD14 antibodies identified an additional subpopulation. Although the majority of cells expressed CD14 as well as CD68 markers, we also detected a population expressing only CD68, properly identifying another SVF-cell subtype (Figure 4A). Indeed, staining of peripheral blood using MQ marker showed that 0.5–1.5% of CD45^+^ cells were macrophages. By contrast, CD68^+^CD14^+^ double-positive cells were in the range of 3–15% due to the fact that CD68 can be also expressed by monocytes (Figure S2).

## 3. Discussion

This study aimed to decipher the morphological and immunological differences of human subcutaneous fat layers, focusing on freshly isolated primary adipocytes as well as adipose-derived stem cells and infiltrating immune cells.

Previous studies have already described morphological and physiological differences of these subcutaneous fat layers, but few of them have focused on the immune contexture within them [15,16].

Herein, we confirmed previous findings by showing that adipocytes from the superficial fat layer significantly differ in size from adipocytes of the deep fat layer. Moreover, we also validated that ASC isolated from SAT proliferated faster and had a higher potential to differentiate into adipocytes than those isolated from DAT. These differences were also detectable on molecular level, which offers the possibility to speculate on the regulatory molecular mechanisms responsible for this phenomenon and draw a conclusion about the precise anatomical function. Since we did not find significant differences in total cellularity of the SVF, we speculated either the existence of an undefined ASC subpopulation or microenvironmental cues that become genomically manifested because of their anatomical origin. The second possibility is of special interest, since a recent study investigating the regulation of regenerative cycles of ASC in dermal white adipose tissue (dWAT) of mice [17] showed that ASC self-renewal and proliferation in mouse dWAT is controlled by PDGFA-dependent regulation of PI3K/AKT2 and correlates with the hypermorphic nature of murine dWAT [18,19,20]. Since human SCAT lacks a defined intradermal fat layer, hair follicle regeneration happens within “cone-like structures” in the most superficial part of SAT. Thus, ASC localized in near proximity to hair follicles account to the human SAT layer and might represent this pool of cells with high regenerative potential. In line with this evidence, we found increased AKT phosphorylation in SAT-ASC.

Besides the spatial proximity to hair follicle cells, other microenvironmental cues may account for the observed differences in the regenerative potential of SAT-ASC. In addition to niche-defining components, such as extracellular matrix composition [21], or systemic active compounds, such as Vitamin D [22], another important regulatory factor is represented by immune cells that either directly (clearance of apoptotic cells) or indirectly (endo-/paracrine activity) might affect ASC phenotype. Immune cells account for about 1/3 of all SVF cells and CD3^+^ T-cells as well as macrophages can be detected in SCAT-SVF [23].

Flow cytometry analysis of T-cells showed no significant differences between SAT and DAT samples, although the proportion of CD3^+^ T-cells compared to peripheral blood was significantly lower. More interestingly, we found an increase (compared to peripheral blood) of mature macrophages in the fat tissue in general and in the SAT layer in particular.

In general, macrophages account for 10–15% of SVF in visceral adipose tissues (VAT) [24], and this amount can increase up to 40–50% in SVF isolated from VAT of obese humans and in obesity mouse models [25]. Independent from the used marker, we found that macrophages preferentially infiltrate SAT since their frequency was increased in SAT in comparison with DAT. Our finding that CD68^+^ macrophages are enriched in SAT has to be discussed in more detail. Increased levels of CD68-mRNA have already been described in a previous study; however, that study reported higher CD68 levels in DAT instead of SAT [26]. This supposed discrepancy might be resolved by the fact that CD68 (together with CD14) is not exclusively enriched in macrophages only, but significant levels might be detected in non-macrophage cell types, such as fibroblasts, preadipocytes, or even adipocytes [27]. To strengthen our findings, we therefore performed stainings using an additional MQ marker, which has been shown to be specific for mature macrophages and is not found on monocytes [14]. Using both marker combinations showed an increase of mature macrophage infiltration in SAT over DAT, also suggesting that determination of increased CD68 expression on its own might be not sufficient to clearly identify macrophages.

Discussing possible reasons for increased macrophage infiltration into SAT, the spatial proximity of SAT to the microbiota of the skin as well as bacteria that can occasionally be found in the fat tissue most proximal to the deep dermal layer [28] might trigger the number and maturation of tissue-resident macrophages. Moreover, it would be interesting to solve the question of whether the accumulation of macrophages in SAT results from increased migration of monocytes from blood or local proliferation of resident macrophages. At least in obesity, macrophage accumulation in adipose tissue is promoted by in situ proliferation of resident macrophages in adipose tissue [29].

It would be interesting to further characterize the phenotype of infiltrated macrophages in SAT and DAT that might be beneficial in the future for the development of novel ASC-based therapeutic approaches in a clinical setting. This has become even more relevant since recent studies showed that M1-polarized macrophages predominate in inflamed subcutaneous tissue of non-healing wounds [30]. On the contrary, ASC-cytokines induced an M2-like macrophage phenotype in vitro, and in vivo the beneficial effects of a combined macrophage/ASC treatment were demonstrated in a mouse model [31]. These findings would suggest a combined macrophages/ASC cell therapy also for non-healing wounds, such as ulcers and burn injuries.

In conclusion, we could show that the origin of subcutaneous adipose-derived stem cells correlates with their regenerative capacity and the provided data indicate that proximity to the skin might promote macrophage infiltration.

## 4. Materials and Methods

### 4.1. Tissue Sampling and Isolation of Cells

Primary adipocytes and ASC were isolated from subcutaneous abdominal fat tissue obtained from a total of 14 female patients (mean ± standard error of the mean (SEM), age 43.6 ± 12.1 years; body mass index (BMI) 25.1 ± 2.3 kg/m^2^; average weight loss 42.8 ± 18.6 kg) undergoing elective abdominoplasty. This study protocol was approved by the Ethics Committee of the Medical University of Innsbruck (EK 301/4.5 and 362/5.2; 10 June 2016)). Written informed consent was obtained from all donors. Not all analyses have been performed for all of the patients; however, SAT and DAT comparison was always conducted from the same patient. Number of analysed patients per investigated parameter is indicated in each figure legend.

Minced pieces of superficial and profound subcutaneous fat tissue were washed with PBS, incubated with collagenase Type I (0.15% in PBS, Worthington, Vienna, Austria) for 1 h at 37 °C, strained using a 200-µm strainer, and incubated at room temperature (RT) for 10 min to separate adipocytes from residual cells. The upper phase containing primary adipocytes was transferred into a new tube and extensively washed with prewarmed PBS. Purified adipocytes were immediately lysed in Trizol-Reagent for RNA isolation or subjected to microscopy to assess viability, purity, and size of adipocytes.

The lower phase containing ASC and other cells from the stromal vascular fraction were centrifuged at 500× *g* for 10 min, treated with erythrocyte lysis buffer (RBC Lysis Buffer (1×), Biolegend, Vienna, Austria) for 20 min at RT, and spun at 500× *g* for 5 min. The stromal vascular fraction was resuspended in DMEM/F12 medium (PAN Biotech, Aidenbach, Germany), filtered through 100 µm and 40 µm nylon mesh cell strainers (VWR, Vienna, Austria), counted with a CASY cell counter (Schärfe System, Reutlingen, Germany), and either subjected to immunostaining for flow cytometry or plated at a density of 10^3^ cells/cm^2^ for culture in PM4 medium [32] containing DMEM/F12 supplemented with 1ng/mL rhFGF2, 10 ng/mL EGF (Immunotools, Friesoythe, Germany), 500 ng/mL Insulin (Roche, Vienna, Austria), 2.5% fetal bovine serum (FBS) (PAN Biotech, Aidenbach, Germany), and 1% Penicillin/Streptomycin (PAN Biotech, Aidenbach, Germany). One hour after plating, non-adherent cells were washed off and attached cells were cultured in PM4 for in vitro analysis.

### 4.2. Ultrasound and Adipocyte Size Determination

Ultrasonography (US) of the abdominal fat tissue was performed on a Philips iU22 device (Philips, Bothell, WA, USA) using a broadband curved-array transducer.

Images of adipocytes from the superficial (SAT) and the profound (DAT) fat layers were acquired using the Olympus CK2 microscope equipped with a JenOptik ProGres CT3 camera controlled by the ProgRes Capture Pro software (version 2.8.9.3, Jenoptik, Jena, Germany). Adipocyte size (diameter, in μm) was determined using ImageJ (version 1.50i, NIH, Bethesda, MD, USA). The measurements were performed by an operator blinded to the origin of the tissue.

### 4.3. Immunohistochemistry and Immunoblotting

The immunohistochemical staining technique of fat tissue samples was described elsewhere [21,33]. In short, paraformaldehyde-fixed tissues (4.5% formaldehyde) were dehydrated (35 min in 100% ethanol, 70 min in isopropyl alcohol, 90 min in paraffin) and embedded in paraffin. Consequently, 3-µm sections were prepared from fixed SAT and DAT tissues, slides were deparaffinised, and a Haematoxylin Eosin staining (Merck, Vienna, Austria) was performed as described by the manufacturer. Image acquisition was conducted with a Zeiss AXIO Imager Z2 microscope (Zeiss, Vienna, Austria). For immunoblotting, 1 × 10^5^ to 5 × 10^5^ cells were lysed in laemmli buffer containing 5% 2-β-mercaptoethanol (Sigma Aldrich, Vienna, Austria), sonicated, and boiled for 5 min at 95 °C. Proteins were size fractioned on prestained gradient polyacrylamide gels (Mini-PROTEAN^®^TGX Stain-Free™ Precast Gels, Bio-Rad, Munich, Germany), blotted onto 0.2 µm PVDF membrane, blocked for 2 h in 7.5% low-fat milk powder, and incubated overnight with primary antibodies against phAKT (Ser473), AKT, php44/42 (Trh202/Tyr204), p44/42, phmTOR (Ser2448), mTOR, ACC, LIPIN1, PLIN1, FABP4, and GAPDH (all obtained from Cell signaling, Leiden, The Netherlands). After washing, horseradish peroxidase conjugated sheep-anti-mouse and sheep-anti-rabbit antibodies were incubated for 1 h and the reaction was visualized by enhanced chemiluminescence reagent ECL (Bio-Rad) using a Bio-Rad ChemidocMP gel analyzer for detection. Quantification was carried out using the ImageLab 5.0 software (version 5.0, Bio-Rad, Munich, Germany) according to the manufacturer’s instructions.

### 4.4. Cell Proliferation and In Vitro Differentiation

To investigate the proliferation and viability of SAT- and DAT-ASC, 5000 cells/well were plated in 96-well plates (Falcon ^®^ 96-well plates, Corning, Austria) and cultured for 6 days in PM4 medium. Medium was exchanged every other day. Cell viability and proliferation were assessed by PrestoBlue cell viability Reagent (analysing mitochondrial activity) and a CyQUANT-cell proliferation assay kit (DNA content) according to the manufacturer’s instructions (Thermo Fisher Scientific, Vienna, Austria).

For in vitro differentiation, 20,000 cells/96-well were plated 24 h before addition of the adipogenesis-inducing medium (AIM) based on DMEM/F12 supplemented with 2.5% FBS, 1 µM Insulin (obtained from Roche, Austria), 100 nM Dexamethasone, 1 µM Troglitazone (TROG), 500 µM 3-isobutyl-1-methyl-xanthine (IBMX), and 250 nM 3,3′,5-Triiodo-l-thyronine (T3). After 7 days, the medium was replaced by adipogenesis determination medium (ADM) consisting of DMEM/F12 supplemented with 2.5% FBS, 1 µM Insulin, and 1 µM TROG for an additional 7 days. The medium was changed twice per week. At day 14, the extent of differentiation was determined by microscopy and flow cytometry of BODIPY™ 493/503 (Thermo Fisher Scientific)-stained cells. For this, BODIPY™ 493/503 (4,4-Difluoro-1,3,5,7,8-Pentamethyl-4-Bora-3a,4a-Diaza-s-Indacene D3922 Thermo Fischer Scientific) was diluted in ethanol at a concentration of 1 mg/mL and differentiated cells were stained at a final concentration of 1 µg/mL for 20 min at 37 °C and subsequently subjected to analysis. Reagents were purchased from Sigma-Aldrich, Austria unless stated otherwise. All experiments were performed in technical triplicates.

### 4.5. RNA Isolation and Quantitative RT-PCR (qPCR)

RNA from SAT and DAT adipocytes was isolated using Trizol-Reagent (MRC Inc. Cincinnati, OH, USA) and cDNA was synthesized using random hexamer primers and an iScript cDNA-synthesis kit (Bio-Rad) as previously described [21]. The qPCR reactions were performed using the SsoAdvanced™ Universal SYBR^®^ Green Supermix kit (Bio-Rad) on a CFX96-qPCR machine (Bio-Rad) using the following protocol: 95 °C for 2 min, 40 cycles of 95 °C (15 s), 60 °C (15 s), and 72 °C (10 s). Gene expression was determined by using the Bio-Rad CFX Manager 3.1 software and CT values were normalized to the mean expression of the three reference genes 18sRNA, Glucuronidase Beta (GUSB), and Glyceraldehyde 3-phosphate dehydrogenase (GAPDH). Real time analysis was in technical duplicates. The referenced and newly designed primers used in this study were synthesized by Microsynth Austria (Table 1) and specificity was tested by the assessment of the melting curve.

### 4.6. Blood

Peripheral blood mononuclear cells (PBMC) were isolated from whole blood using Lymphoprep (Axis-Shield, Oslo, Norway) as described previously [38]. In brief, 10 mL of blood were mixed 1:2 with PBS and layered on Lymphoprep. After centrifugation and washing steps, cells were resuspended in PBS with 3% FBS for immunostaining and flow cytometry analysis.

### 4.7. Flow Cytometry Analysis

PBMC isolated from blood and SVF from SAT and DAT were resuspended in PBS with 3% FBS for labelling. To discriminate between live and dead cells, cells were stained with the Fixable Viability Dye eFluor^®^ 450 (Thermo Fisher Scientific). Endothelial progenitors (EPC) and adipose stem cells (ASC) were stained with monoclonal antibodies against the following surface markers: CD45 (clone HI30), CD31 (WM-59), CD34 (561) (all Biolegend, Koblenz, Germany), and CD90 (eBio5E10) (Thermo Fisher Scientific, Vienna, Austria). T-cells were stained with monoclonal antibodies against the following surface markers: CD45 (HI30) (Thermo Fisher Scientific Vienna, Austria), CD3 (SP34-2), and CD8 (Sk1) (BD Biosciences, Vienna, Austria). Macrophages were stained with monoclonal antibodies against the following surface markers: CD14 (61D3), CD45 (HI30), and MQ(25f9) (Thermo Fisher Scientific, Vienna, Austria). For intracellular CD68 staining, cells were permeabilized using the FIX & PERM Cell permeabilization kit according the manufacturer’s instructions and stained with anti-CD68 antibody (Y1/82A) (Biolegend, Koblenz, Germany). Finally, cells were acquired on a BD LSRFortessa™ flow cytometer using DIVA software (BD Biosciences, San Jose, CA, USA). Results were analyzed using FlowJo software (TreeStar, Ashland, OR, USA). The gating strategy is shown in Figure 4A. Moreover, gating was also made according to the fluorescence minus one (FMO), where cells were stained with all antibodies except the one of interest.

### 4.8. Data Analysis

Statistical analysis was performed in R (https://r-project.org) version 3.4.3. To compare the means, a paired *t*-test or the Student’s *t*-test was used. The data are shown as mean ± SD. Differences were considered to be significant at *p* < 0.05.

## Figures and Tables

**Table 1:** Primer pairs used for mRNA determination.

Gene	Sense Primer	Antisense Primer
human Leptin	5′-CACACGCAGTCAGTCTCCTC-3′	5′-AGGTTCTCCAGGTCGTTGG-3′
human ADIPOQ	5′-GATGGCAGAGATGGCACCC-3′	5′-GGAATTTACCAGTGGAGCCA-3′
human RBP4	5′-TTCGACAAGGCTCGCTTCTC-3′	5′-CGATGTTGTCCTGCAGAAAGAG-3′
human CMKLR [34]	5′-TGGAAGAAACCCGAGTGCAAA-3′	5′-AGAACTTGGGTCTCTATGGGG-3′
human DEFB1 [35]	5′-CCAGTCGCCATGAGAACTTCC-3′	5′-GTGAGAAAGTTACCACCTGAGGC-3′
human NAMPT	5′-GCAGAAGCCGAGTTCAACAT-3′	5′-TCTGTCTTCTTTTCACGGCA-3′
human MCP1 [36]	5′-GTCTTGAAGATCACAGCTTCTTTG-3′	5′-AGCCAGATGCAATCAATGCC-3′
human MCSF	5‘-GCAGCTGCAGGAACTCTCTT-3′	5‘-CCAGCAACTGGAGAGGTGTC-3′
human 18sRNA [37]	5′-GCAATTATTCCCCATGAACG-3′	5′-GGCCTCACTAAACCATCCAA-3′
human GUSB	5′-GGAATTTTGCCGATTTCATGAC-3′	5′-TCTCTGCCGAGTGAAGATCCC-3′
human GAPDH	5′-CAACGAATTTACAGCA-3′	5′-TGTGAGGAGGATTCAG-3′

## Supplementary Materials

Supplementary materials can be found at http://www.mdpi.com/1422-0067/19/5/1404/s1.

## Abbreviations

ASC	Adipose derived stem cell
SAT	Superficial adipose tissue
DAT	Deep adipose tissue
SCAT	Subcutaneous adipose tissue
SVF	Stromal vascular fraction
