# Supplementary material for: Human Macrophages Preferentially Infiltrate the Superficial Adipose Tissue
Source: Int J Mol Sci. 2018 May 8;19(5):1404. doi: 10.3390/ijms19051404 (PMC5983635; doi:10.3390/ijms19051404)
Supplement: Supplementary file 1 [file ijms-19-01404-s001.pdf]

Supplementary Materials

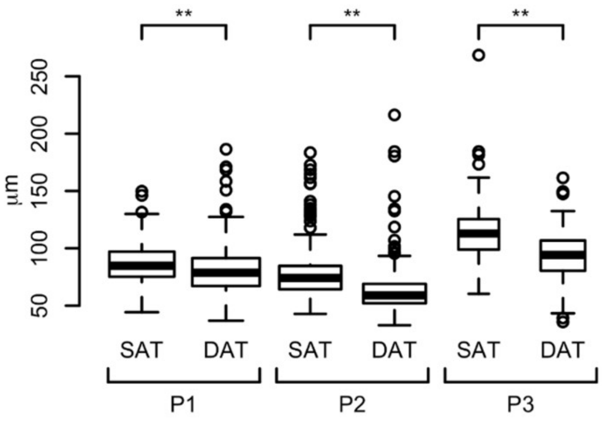

Figure S1. Quantitative analyses of freshly isolated adipocytes from SAT or DAT in each individual patient. The box plot represents data from a total of 2167 adipocytes isolated from each patient. Significance for difference of the means was calculated using a paired t-test (** *p*-value < 0.01).

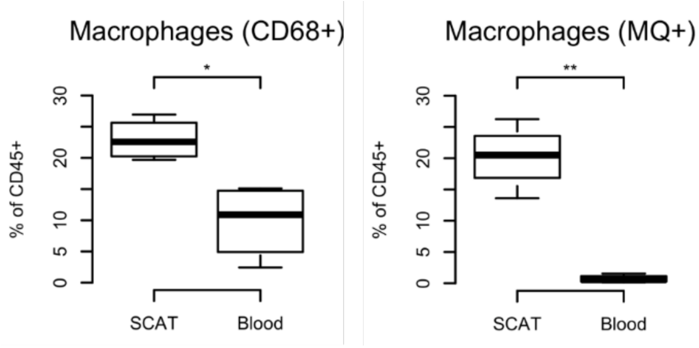

Figure S2. Macrophage staining with MQ (clone 25F9) and CD68 markers in fat adipose tissue (SAT and DAT) and blood. Thus, this figure includes data from paired samples (SVF and PB). Cells were gated on $CD45^+$ viable cells. Macrophage (MΦ) ($CD14^+$ $CD68^+$ or $CD14^+$ MQ^+) are shown as % of $CD45^+$ cells. Results represent data from four patients and are expressed as mean ± SD. Significance of the difference in means was assessed using the Student's *t*-test (* *p*-value < 0.05, ** *p*-value < 0.01).
